# A fundus image dataset for intelligent retinopathy of prematurity system

**DOI:** 10.1038/s41597-024-03362-5

**Published:** 2024-05-27

**Authors:** Xinyu Zhao, Shaobin Chen, Sifan Zhang, Yaling Liu, Yarou Hu, Duo Yuan, Liqiong Xie, Xiayuan Luo, Mianying Zheng, Ruyin Tian, Yi Chen, Tao Tan, Zhen Yu, Yue Sun, Zhenquan Wu, Guoming Zhang

**Affiliations:** 1 Shenzhen Eye Hospital, Jinan University, Shenzhen Eye Institute, Shenzhen, China; 2 Faculty of Applied Sciences, Macao Polytechnic University, Macao Special Administrative Region of China, Macao, China; 3 Department of Biology, New York University, New York, NY US; 4 State Key Laboratory of Ophthalmology, Zhongshan Ophthalmic Center, Sun Yat-sen University, Guangzhou, 510060 China

**Keywords:** Computational biology and bioinformatics, Retinal diseases

## Abstract

Image-based artificial intelligence (AI) systems stand as the major modality for evaluating ophthalmic conditions. However, most of the currently available AI systems are designed for experimental research using single-central datasets. Most of them fell short of application in real-world clinical settings. In this study, we collected a dataset of 1,099 fundus images in both normal and pathologic eyes from 483 premature infants for intelligent retinopathy of prematurity (ROP) system development and validation. Dataset diversity was visualized with a spatial scatter plot. Image classification was conducted by three annotators. To the best of our knowledge, this is one of the largest fundus datasets on ROP, and we believe it is conducive to the real-world application of AI systems.

## Background & Summary

Retinopathy of prematurity (ROP), a retinal vascular disorder in preterm infants, stands as one of the leading causes of blindness in the pediatric population^1^. Its severity is indicated by five stages, from stage 1 (demarcation line) to stage 5 (total retinal detachment)^1^. Most of the ROP cases are mild and can regress spontaneously, thus the actual cases requiring treatment are rare. However, once the conditions are identified as treatment-required, they should be intervened timely and appropriately to prevent retinal detachment with consequent blindness^2,3^.

A fundus imaging system offers the potential to accurately and efficiently identify cases that require timely treatment, as fundus images can be used to record the severity and progress of the disease objectively and with high sensitivity and specificity. Therefore, fundus imaging systems are most widely used for ROP screening and in-depth analysis^3^.

The importance and applicability of retinal images in the evaluation of disease severity^4–8^ were underlined by the recent studies that have achieved AI systems capable of automated diagnosis and treatment for ROP. Yet few of them met the standards of real-world application, suggesting further research remains warranted. In addition, the performance of AI models can be much strengthened if fundus images of multi-ethnicities and from multi-centers are used for development. Therefore, the publication of datasets on ROP will lend extra power to the development of AI models, and the validation of AI models already developed.

At present, there are many public datasets of fundus images for adults^9–15^, but few are for infants, meaning available resources suitable for the development and validation of AI models for ROP remain scanty. Therefore, an ideal fundus image dataset for infants is beneficial to bridge the gap in clinical scenarios. The dataset we provided has a relatively large sample size in terms of the medical image classification task for newborn infants. Our team has engaged in clinical work on ROP diagnosis and treatment since 2003 and has accumulated a wealth of clinical data. Related research has been published in ophthalmic journals^16–23^. Since 2018, we have focused on AI systems for retinal diseases and have generated some preliminary research results^7,24–26^. We are concentrating on improving model robustness to realize clinical application. Moreover, we hope that the publication of the ROP dataset will be of use to researchers globally to facilitate computer-aided ROP diagnosis and treatment systems.

**Fig. 1:**
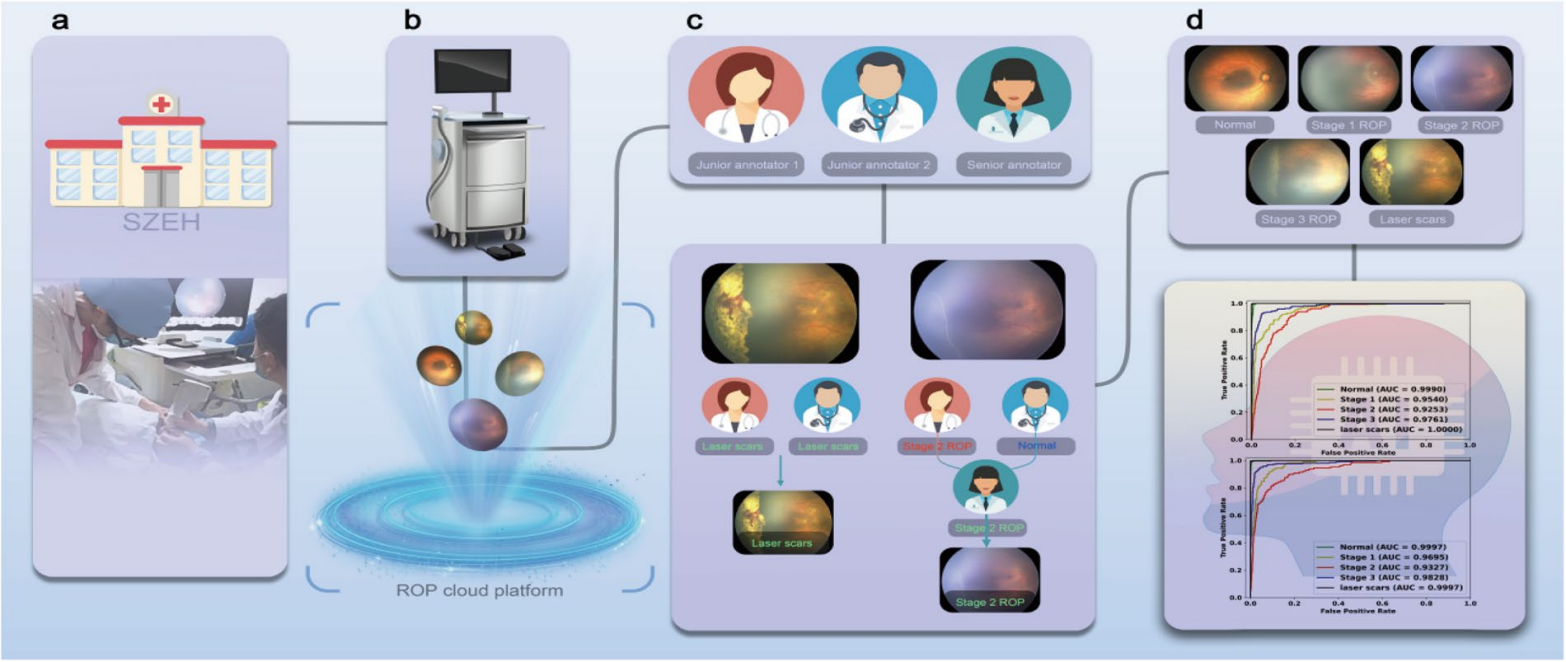
Workflow of the establishment of the ROP dataset. (**a**) Hospitals and scenarios for data collection. (**b**) A total of 1,099 fundus images were collected using the imaging tools and uploaded into the ROP cloud platform. (**c**) Three annotators who had completed guideline-based training and image annotation tests were included in image classification tasks. The annotation process consisted of initial annotation by two junior annotators and further verification by one senior annotator. (**d**) The final classification results were used to develop AI models for automated ROP staging. The AI models achieved excellent performance with AUCs > 0.9.

In this study, we provide numerous fundus images ranging from stages 1 to 3, normal images, and images with laser scars. The brief description of this study is depicted in Fig. 1. We hope this dataset will shed insight to the development of AI models that are more clinically applicable. Finally, AI has the potential to revolutionize the medical industry for future preterm infants. Thus, we will continue to collect and publish relevant clinical data.

## Methods

### Data collection

A total of 1,099 fundus images from 2004 to 2023 were collected in the Shenzhen Eye Hospital (SZEH). Prior to infant examinations, parents of the infants signed consent forms for data usage to assist in scientific research, and all associated images have been anonymized to protect the privacy. This study followed the tenets of the Helsinki Declaration and was approved by the Medical Ethics Committee of SZEH (ID: 2023KYPJ091). Images in this study were captured using three kinds of wide-field contact fundus imaging tools, including the RetCam (RetCam, Clarity Medical Systems, Inc. USA), ROP Screening System (SW-8000, China), and Nautilus fundus imaging system (RS-B002, China). The process for capturing images was as follows: the infants’ pupils were dilated with tropicamide approximately 30 minutes before the examination. Fundus photography was performed by well-trained technicians or ophthalmologists with several assistants^3^. The standard six-orientation images mainly included the optic disc central, macula central, temporal, nasal, superior, and inferior.

We mainly excluded images that were redundant and those with poor quality, leading to unclear depiction of lesions. Finally, we included images with clear imaging of the retina, vessels, and lesions. Image quality is a crucial assessment criterion for our image selection process. The clarity of structures and lesions, including retina, blood vessels, laser scars, ROP lesions, etc., in the images is a prerequisite for inclusion. For normal images, the focal point is on the retina; for images with lesions, the focal point is clearer at the site of the lesion. The brightness of the images we selected falls within a moderate range, excluding images that are too bright or too dark, which would make certain areas unobservable. The integrity of all images included in this study is generally considered clinically acceptable and usable.

Because ROP conditions are progressive, there may be multiple follow-ups for the same infant. In addition, some severe ROP infants have undergone laser therapy. Therefore, the same eye of an infant may include more than one fundus image. Due to variations in image dimensions captured by different devices, we implemented pre-processing techniques. This involved uniformly cropping the images to a resolution of 512 × 512, while preserving the aspect ratio of the retinal region.

### Image categorization

**Fig. 2:**
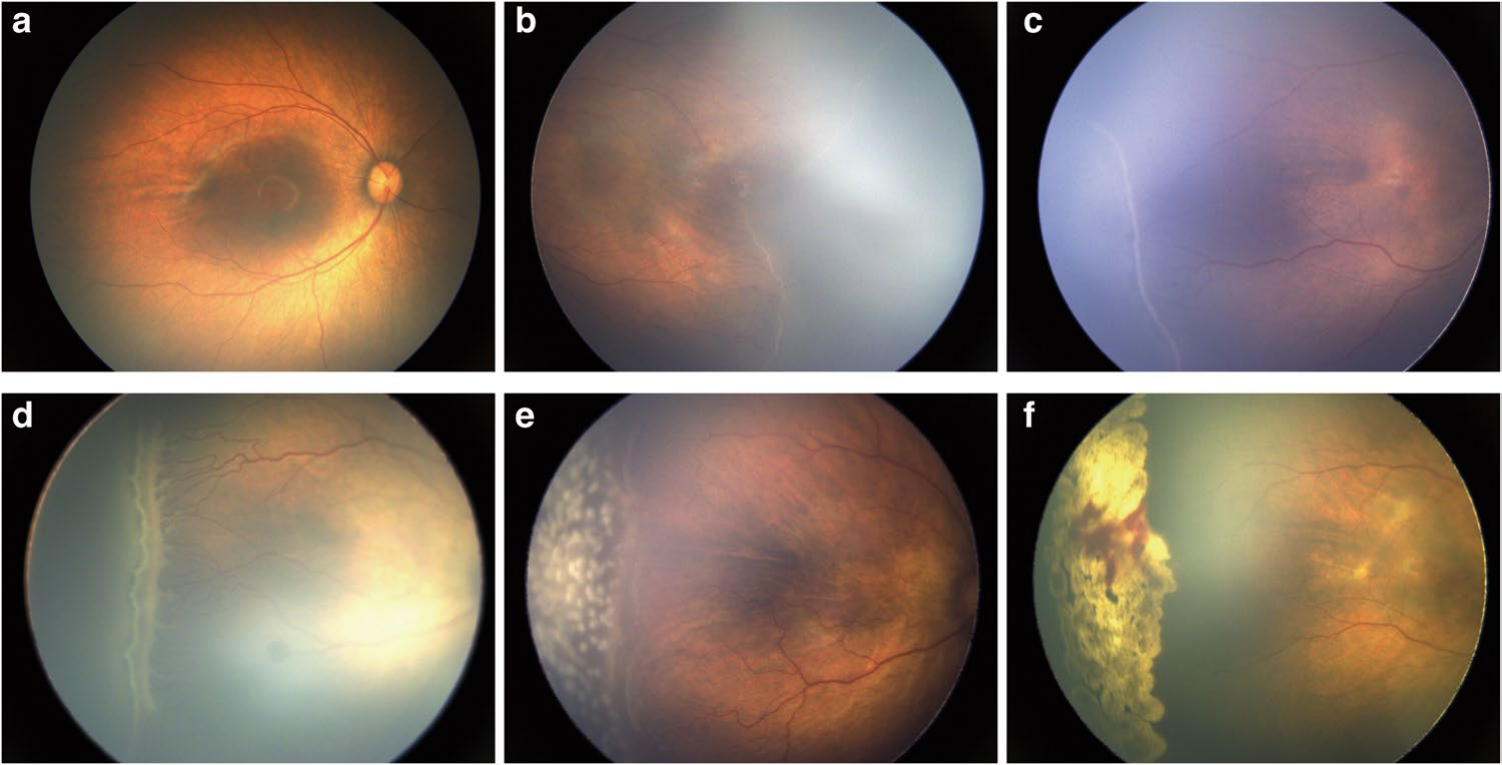
Demonstration of various kinds of fundus images. (**a**), normal image; (**b**), stage 1 ROP; (**c**), stage 2 ROP; (**d**), stage 3 ROP; (**e**), image with fresh laser scars; and (**f**), image with stable laser scars.

According to the International Classification of Retinopathy of Prematurity Third Edition (ICROP)^27^, all fundus images with ROP were classified into 5 stages. Of these, Stages 1 and 2 ROP represent cases of mild ROP, Stage 3 ROP represents treatment-required cases, and Stages 4 and 5 ROP represent late ROP. Clinically, doctors should determine the ROP severity to adopt different treatment strategies. Stages 4 and 5 ROP are rare in clinical settings; therefore, only images of infants with Stages 1 to 3 ROP were included in this study (Fig. 2). The distribution of images for each category is shown in Table 1.

**Table 1:** The distribution of images for five categories.

	Training set	Validation set	Test Set	Total images	Eyes	Infants
Normal	188	24	24	236	215	130
Stage 1	75	9	10	94	87	71
Stage 2	132	16	17	165	138	112
Stage 3	208	26	27	261	227	162
Laser	274	34	35	343	280	163
Total	877	109	113	1099	789	483

Three experienced annotators from the SZEH classified all the images into the aforementioned categories. Specifically, two junior annotators classified all the fundus images independently, and in the condition of discrepancy from two junior annotators, a third annotator with higher seniority re-classified the images with inconsistent classification results. We conducted a detailed analysis on the consistency evaluation between intra-annotators and inter-annotators. The Cohen’s Kappa coefficient between two junior annotators on the same dataset is 0.856, indicating a high consensus between the two annotators’ classification results. In addition, we also evaluated the consistency between two annotators at different time points on the same dataset. The Cohen’s Kappa coefficients between two annotators at different time points on the same dataset are 0.929 and 0.919, respectively. This proves that the annotators have a high consensus at different time points. Therefore, we evaluated the consistency between inter-annotators and intra-annotators through the Cohen’s Kappa coefficient, and the results show a high consensus, which help us further verify and improve the quality and accuracy of the data. It is worth noting that the classification result of each image may not be fully consistent with that by different ophthalmologists around the world even after going through the aforementioned steps. Even if all the ophthalmologists use the same ICROP criteria to classify fundus images^27^, different ophthalmologists may provide different classification results using their visual judgment. Inconsistency of annotators in classifying stage 1 and 2 ROP was most evident.

## Data Records

The dataset is available at Figshare in the form of a zipped file^28^. The zipped file folder primarily contains the fundus images of the included infants. Images are named as “Normal, Stage 1 ROP, Stage 2 ROP, Stage 3 ROP, and Laser scars”, by which “Normal” represents images without any ROP diseases, “Stages 1 to 3” represents images with ROP ranging from Stage 1 to 3, and “Laser scars” represents images with laser scars after laser therapy due to ROP. The majority of our data consists of temporal images, with some including the optic disc, while others with lesion near the periphery do not include the optic disc. For certain infants, whose temporal images had inadequate quality, we selected images with posterior pole or other orientations. The final selection of image orientation was determined by the location and condition of the lesions.

This dataset can be used for different research purposes, such as automated ROP identification, localization, and segmentation of avascular retina in ROP, as well as validation of ROP-related AI models that have been developed by several researchers. The current data classification strategy was conducted by our team using ICROP criteria; therefore, researchers can use and alter it for different research purposes.

**Fig. 3:**
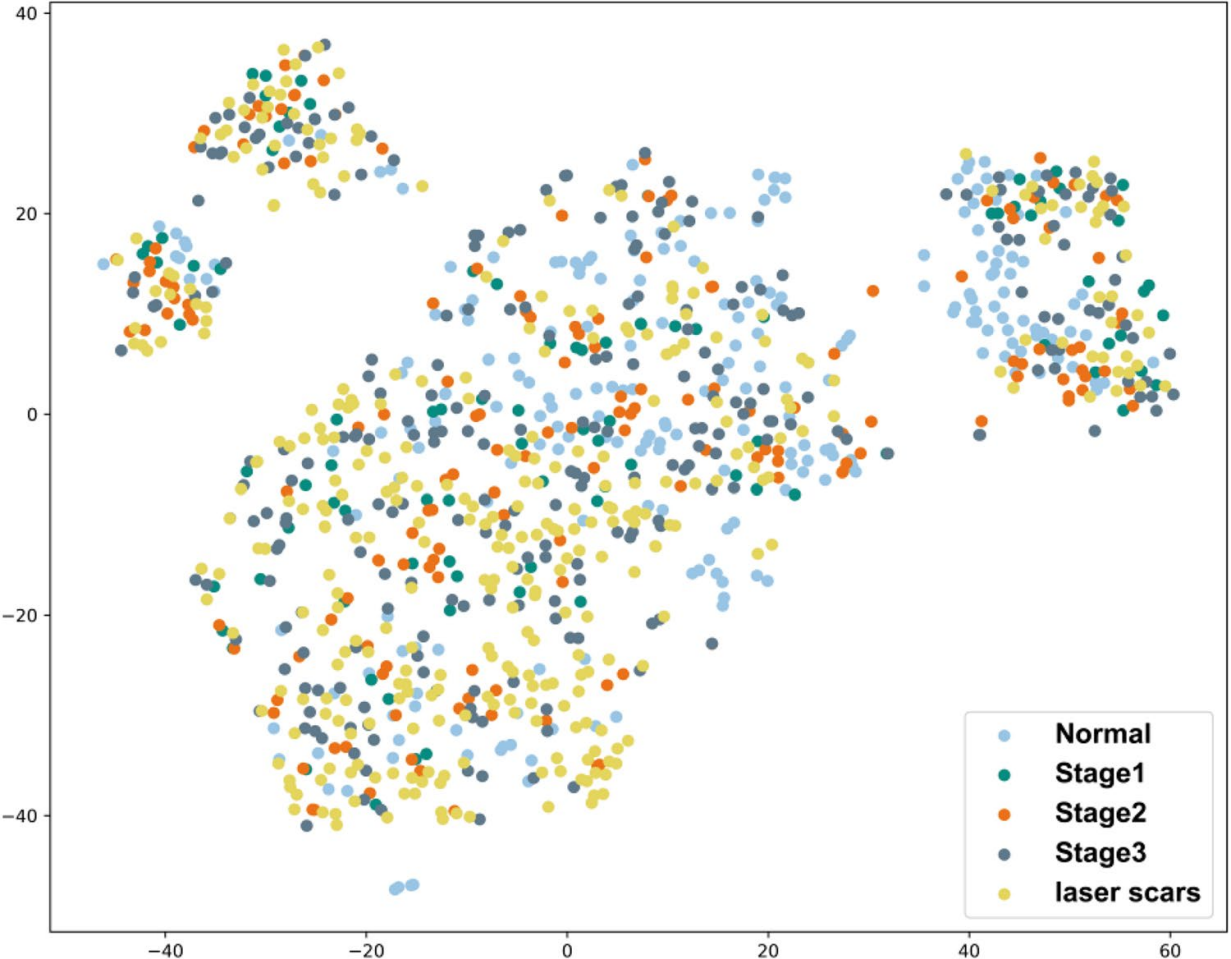
Spatial scatter plot of ROP datasets. When presenting the distribution of different categories in the dataset, high-dimensional image data is mapped to a two-dimensional space using the t-SNE dimensionality reduction technique. This preserves the local structure of the original data, ensuring that similar samples remain close in the reduced-dimensional space. Finally, the mapped two-dimensional data is visualized through a scatter plot.

Detailed descriptions of the ROP dataset are shown in Table 1. To visually observe the distribution characteristics of five different categories of the dataset, we use t-distributed Stochastic Neighbor Embedding (t-SNE)^29^ technique to visualize the features of different categories of the datasets by providing a location in a two-dimensional map for each datapoint of the high-dimensional image feature. The purpose of dimensionality reduction is to retain as much of the significant structure of high-dimensional data as possible in a low-dimensional map. As a tool to visualize high-dimensional data, t-SNE converts the similarity between datapoints of image features to joint probabilities and aims to minimize the Kullback-Leibler divergence between the joint probabilities of the low-dimensional embedding and the high-dimensional data, which can convert high-dimensional dataset into two-dimensional data that can be displayed in a scatter plot^29^. We first use Opencv to read each image data, and then flatten the features of each image into one-dimensional vector spaces. The flattened image features are input into t-SNE for dimensionality reduction. T-SNE can capture most of the local structure of high-dimensional data and can also reveal the global structure. Finally, each image feature will be converted into a point in the two-dimensional map. The points represented by images belonging to the same category are represented by the same color to obtain the scatter plot of ROP datasets as shown in Fig. 3. There are five kinds of symbols in the visualized figure, representing the five subsets.

## Technical Validation

The 1,099 fundus images included in the dataset are obtained from 483 infants, with 36.44% being female. The infants’ birth weights ranged from 1247 ± 432 g, and gestational ages at birth ranged from 29.10 ± 2.62 weeks. All infants included in this study are Asian. A corresponding label of each fundus image is included in the dataset.

**Fig. 4:**
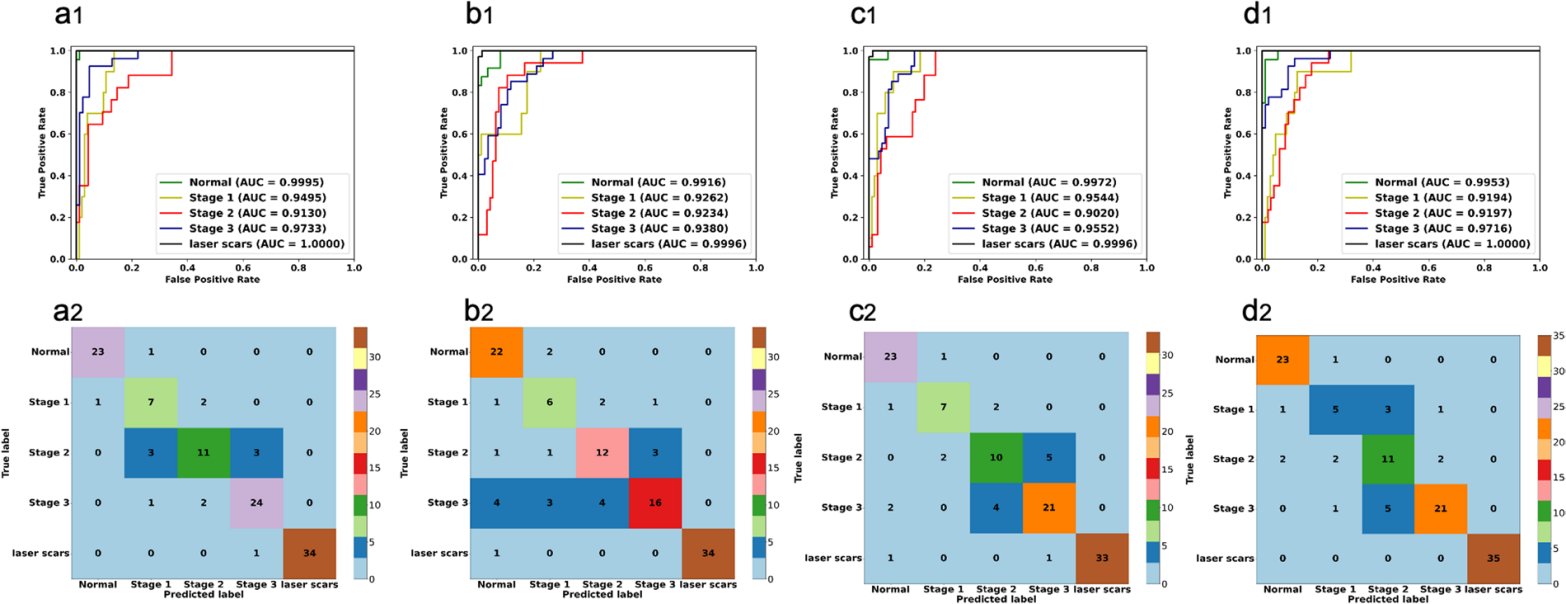
The classification results of included fundus images based on different AI models. (**a**-**d**) display the performance of the ResNet 50 model, ResNet101 model, ConvNeXt-T model, and ViT-B model.

We also developed four automated ROP staging models to automatically identify these fundus images. We divide the dataset into training set, validation set, and test set according to the ratio of 8:1:1. Furthermore, we adopt some common data augmentation strategies, including random crop, random horizontal and vertical flipping, etc. We use stochastic gradient descent to optimize parameters with a learning rate of 1e-3, momentum of 0.9, and weight decay of 1e-4. The batch size is set to 64. All the AI models are implemented on an NVIDIA V100 GPU. The classification results are shown in Table 2. ResNet50^30^, ResNet101^31^, ConvNeXt-T^32^, and ViT-B^33^ were selected as algorithms for AI model development. The classification results in test dataset shows that all the AI models can achieve excellent performance in the classification tasks (Fig. 4, Table 2). Among them, and the AI model developed by ResNet50 achieved the best performance. These results support the technical quality of the ROP dataset.

**Table 2:** The classification results of AI models.

Method	Accuracy	AUC	Precision	Sensitivity	F1 score	Specificity	Kappa
ResNet50	87.61	96.71	82.64	83.31	82.81	97.04	83.97
ResNet101	79.65	95.58	74.51	75.73	74.55	95.08	73.79
ConvNeXt-T	83.19	96.17	79.09	79.34	79.13	95.87	78.24
ViT-B	84.07	96.12	77.88	77.66	77.62	96.18	79.37

## Usage Notes

We encourage users of the data to cite the figshare record, and this article, in any related reports.

## Data Availability

No novel code was used in the construction of ROP dataset.

## References

[1] Stahl A (2022). Effect of intravitreal aflibercept vs laser photocoagulation on treatment success of retinopathy of prematurity: The FIREFLEYE randomized clinical trial. Jama.

[2] Blencowe H, Lawn JE, Vazquez T, Fielder A, Gilbert C (2013). Preterm-associated visual impairment and estimates of retinopathy of prematurity at regional and global levels for 2010. Pediatr Res.

[3] Lin, J. Y. *et al*. Comparison of RetCam and smartphone-based photography for retinopathy of prematurity screening. *Diagnostics (Basel)* **12**, 10.3390/diagnostics12040945 (2022). 10.3390/diagnostics12040945 PMC9029155 35453993

[4] Campbell JP (2021). Evaluation of a deep learning-derived quantitative retinopathy of prematurity severity scale. Ophthalmology.

[5] Campbell JP (2022). Artificial intelligence for retinopathy of prematurity: validation of a vascular severity scale against international expert diagnosis. Ophthalmology.

[6] Coyner AS (2019). Automated fundus image quality assessment in retinopathy of prematurity using deep convolutional neural networks. Ophthalmol Retina.

[7] Xie H (2023). Adversarial learning-based multi-level dense-transmission knowledge distillation for AP-ROP detection. Med Image Anal.

[8] McCourt EA (2018). Validation of the colorado retinopathy of prematurity screening model. JAMA Ophthalmol.

[9] Huang X (2023). GRAPE: A multi-modal dataset of longitudinal follow-up visual field and fundus images for glaucoma management. Sci. Data..

[10] Jin K (2023). MSHF: A multi-source heterogeneous fundus (MSHF) dataset for image quality assessment. Sci. Data..

[11] Jin K (2022). FIVES: A fundus image dataset for artificial intelligence based vessel segmentation. Sci. Data..

[12] Kovalyk O (2022). PAPILA: Dataset with fundus images and clinical data of both eyes of the same patient for glaucoma assessment. Sci. Data..

[13] Kumar JRH (2023). Chákṣu: A glaucoma specific fundus image database. Sci. Data..

[14] Kumar JRH (2023). Author aorrection: Chákṣu: A glaucoma specific fundus image database. Sci. Data..

[15] Lin L (2020). The SUSTech-SYSU dataset for automated exudate detection and diabetic retinopathy grading. Sci. Data..

[16] Hu, X. *et al*. Glim-net: chronic glaucoma forecast transformer for irregularly sampled sequential fundus images. *IEEE Transactions on Medical Imaging*, 1875-1884 (2023). 10.1109/TMI.2023.3243692 37022815

[17] Wu Z (2022). Comparison of clinical outcomes of conbercept versus ranibizumab treatment for retinopathy of prematurity: a multicentral prospective randomised controlled trial. Br J Ophthalmol.

[18] Zhao J (2020). Comparison of OCT angiography in children with a history of intravitreal injection of ranibizumab versus laser photocoagulation for retinopathy of prematurity. Br J Ophthalmol.

[19] Hu Y (2023). Refractive status and biometric characteristics of children with familial exudative vitreoretinopathy. Invest Ophthalmol Vis Sci.

[20] Fan Z (2023). Awareness, prevalence, and knowledge of dry eye among Internet professionals: a cross-sectional study in China. Eye Contact Lens.

[21] Lu X (2022). Refractive and biometrical characteristics of children with retinopathy of prematurity who received laser photocoagulation or intravitreal ranibizumab injection. Graefes Arch Clin Exp Ophthalmol.

[22] Yang Y (2020). Targeted blood metabolomic study on retinopathy of prematurity. Invest Ophthalmol Vis Sci.

[23] Yang Y (2022). Comparative analysis reveals novel changes in plasma metabolites and metabolomic networks of infants with retinopathy of prematurity. Invest Ophthalmol Vis Sci.

[24] Zhang, Y. *et al*. Development of an automated screening system for retinopathy of prematurity using a deep neural network for wide-angle retinal images. *IEEE Access* (2018).

[25] R Zhang *et al*. Automatic diagnosis for aggressive posterior petinopathy of prematurity via deep attentive convolutional neural network. *Expert Systems with Applications* (2021).

[26] Zhao J, Lei, B., Wu, Z., Zhang, Y. & Zhang, G. A deep learning framework for identifying Zone I in RetCam images. *IEEE Access* **PP**, 1-1 (2019).

[27] Chiang MF (2021). International classification of retinopathy of prematurity, third edition. Ophthalmology.

[28] Zhao XC (2024). figshare.

[29] Van der Maaten L, Hinton G (2008). Visualizing data using t-SNE. Journal of machine learning research.

[30] He, K. M., Zhang, X. Y., Ren, S. Q., Sun, J. & Ieee. in *2016 IEEE CONFERENCE ON COMPUTER VISION AND PATTERN RECOGNITION (CVPR)*. 770-778 (IEEE Comp Soc, 2016).

[31] He, K., Zhang, X., Ren, S. & Sun, J. in *Proceedings of the IEEE conference on computer vision and pattern recognition*. 770-778.

[32] Liu, Z. *et al*. in *2022 IEEE/CVF CONFERENCE ON COMPUTER VISION AND PATTERN RECOGNITION (CVPR)*. 11966-11976 (IEEE COMPUTER SOC, 2022).

[33] Dosovitskiy, A. *et al*. An image is worth 16x16 words: Transformers for image recognition at scale. *arXiv preprint arXiv:2010.11929* (2020).

